# Consumption of sweetened-beverages and poverty in Colombia: when access is not an advantage

**DOI:** 10.1186/s12889-018-5037-1

**Published:** 2018-01-15

**Authors:** Oscar F. Herran, Gonzalo A. Patiño, Edna M. Gamboa

**Affiliations:** 1 Universidad Industrial de Santander, Carrera 32 No. 29-31, 680002 Bucaramanga, Santander Colombia; 2 Escuela de Nutrición y Dietética, Wolfville, Canada; 3 Escuela de Administración y Economía, Sulaimani, Iraq

**Keywords:** Health inequalities, Sugar-sweetened beverages, Nutrition surveys, Colombia

## Abstract

**Background:**

This study characterizes the intake of sweetened beverages and establishes whether economic inequalities in their consumption exists.

**Methods:**

Ecological study. Mixed methods using food frequency questionnaire and inequality indices. Based on the National Nutrition Survey, Colombia, 2010. The sweetened beverage intake of 17,514 subjects in 33 geodemographic units was estimated with a food frequency questionnaire and summarized. The calculation of inequality was based on the monetary poverty. The prevalence (yes/no) and frequency (times/day) of sweetened beverage consumption were estimated. Indices of economic inequality were calculated for both prevalence and frequency.

**Results:**

The prevalence of sweetened beverage consumption was between 79.2% (95% CI, 75.7 to 82.8) in adults and 88.5% (95% CI, 85.8 to 91.3) in minors. The frequency of consumption in terms of times/day, was between 0.20 (95% CI, 0.16 to 0.24) in adults and 0.40 (95% CI, 0.33 to 0.46) in minors. The Gini coefficient for the prevalence was close to zero, between 0.04 and 0.08; for the frequency, it was slightly higher, between 0.12 and 0.25.

**Conclusions:**

It was established that there is no economic inequality in the consumption of sweetened beverages. Consumption taxes could be regressive.

**Electronic supplementary material:**

The online version of this article (10.1186/s12889-018-5037-1) contains supplementary material, which is available to authorized users.

## Background

Colombia, like all countries in the Americas, has experienced changes in food consumption patterns that occur in a context of economic growth but have profound social and economic inequalities [1–3]. In middle- and low-income countries, the incidence of overweight in children and adults has increased steadily over the past 20 years, and health-related nutritional influences (or nutritional transitions) are ongoing [4–9]. The probable causes of this phenomenon, which, in turn, leads to an increase in chronic diseases, are located from the individual to the ecological level [10].

The causes of the increase in weight throughout the population include physical inactivity, the food environment, food availability, unbalanced food consumption, poverty, inequality, food insecurity, low parental education levels, and other variables, including cultural variables, and their effects are difficult to isolate [11, 12]. The regulation of sweetened beverages (SB), including sugar-sweetened beverages (SSB), has been the subject of public policy [13, 14]. Some countries, including some in the Americas, have incorporated a tax on SSB consumption, whereas in other countries, such as Colombia, there are ongoing debates surrounding taxation [14–16].

There is scarce literature describing the economic inequalities in SSB consumption specifically and in the consumption of SB in general. Studies of SSB and SB consumption disparities have been conducted in developed countries, where nutritional and food transitions are extreme, and have centered on the differences among individuals in terms of income level, sex, and race [17, 18]. In Latin America, the closest approximations of economic inequality have been made to evaluate the impact of taxes on the consumption of SSB [19]. This study presents a distinct perspective; it was conducted in a middle-income developing country where nutritional and food transitions are ongoing and was performed at the ecological level, in the classical epidemiological sense, to examine the relationship between SB consumption and monetary poverty (%). In addition, the measurement challenges involved in establishing public policies, including those related to consumption taxes, are discussed. Given that the analysis of inequalities often concentrates on the socioeconomic dimension, this analysis should be considered in terms of “economic disparities in nutrition” [20–24]. The question that this study aims to answer is whether, at the ecological level, there are economic inequalities in the consumption of SB by age and sex. The hypothesis is that at least in theory, disadvantages and inequalities exist for women and children and for the poorest groups, and these should be reflected in the consumption of SB [13–19].

## Methods

This study is classified as ecological and is based on data from the National Nutrition Situation Survey (ENSIN) in 2010 [25] and on monetary poverty (%) in 33 geodemographic units of Colombia. The level of analysis is ecological since the variables studied were individual measurements that were then aggregated to represent the unit of analysis, which is the geodemographic unit.

### Study population

In ENSIN-2010, participants were selected through multi-stage stratified sampling to represent 99% of the country’s population. All municipalities of the 32 departments and the capital of the country (geodemographic units) were grouped into strata with similar sociodemographic characteristics. The survey included 50,670 households, in which 10,187 subjects aged 5 to 17 years and 7327 subjects aged 18 to 64 years completed a Food Frequency Questionnaire (FFQ). Based on the questionnaire responses, the consumption of SB was estimated for each of the 33 geodemographic units.

### Sample (n)

The SB intake of 17,514 subjects in 33 geodemographic units was summarized.

### Information sources

For this analysis, we studied a nutritional variable, SB consumption, in subjects of both sexes between 5 and 64 years of age in relation to an economic type, monetary poverty (%). The information on monetary poverty (%) was estimated and reported by the National Administrative Department of Statistics (DANE) for each geodemographic unit in 2011 (the closest year to the ENSIN-2010 data) [26]. Nine of the 33 geodemographic units did not have information regarding their monetary poverty and were excluded from some calculations. Data on the size of the population size between 5 and 64 years of age by sex in the geodemographic units were obtained from population and birth projections by area and sex for the year 2010; this information is reported by the DANE and is available on its website.

### Variables studied

#### Measurement of consumption: prevalence and frequency of SB consumption

In Colombia, ENSINs have been carried out since 2005. Every five years, these surveys study the state of nutrition through anthropometric and biochemical measurements. For the ENSIN-2010, an FFQ was used to study the food and nutrition practices of interest to public health. The checklist of foods, food groups and practices was designed by nutritionists based on the food consumption and nutrition problems identified in the ENSIN-2005. The response section was adapted from two successful reproducibility and validity studies of FFQs used in the Colombian population [27, 28]. Before the FFQ used in the ENSIN-2010 was approved, the facial validity of all its items was guaranteed by applying and adjusting the items in successive pilot tests in the field.

The ENSIN-2010 estimated the frequency of consumption of 30 foods or food groups and three related practices based on an FFQ that offered 10 response categories indicating consumption/practices in the previous month. The applied FFQ did not differentiate the consumption of SSB or SB; it included both in the same item. In this study, the item “*In a typical month, how often do you consume SB (boxed, powdered, bottled)*” was analyzed through two expressions of consumption: the prevalence (yes/no) and frequency (number of occasions per day that SB were consumed: frequency/day or times/day). The frequency responses in the FFQ were converted into a continuous variable (times/day) using appropriate dividers to express the frequency of consumption in units of “times/day” [25]. The prevalence (%) and frequency/day was calculated for each of the geodemographic units for men and women between 5 and 64 years of age, except for pregnant women and subjects who were following diets prescribed by nutritionists or general physicians. The FFQ was administered in the subjects’ homes by nutritionist-dietitians through direct interview. The respondent’s privacy was guaranteed throughout the interview. The nutritionists were trained in the interview methods and in applying the FFQ for one month. All the interviews were followed the same procedures, which were standardized and guaranteed in the training. When the FFQ was administered to children between 5 and 11 years of age, their mothers or caregivers responded. The Colombian Institute of Family Welfare (ICBF) obtained informed consent from the participants prior to enrollment.

#### Statistical analysis

The analysis aimed to identify inequality in SB consumption (prevalence and frequency/day) using monetary poverty (%) as the economic variable [28]. The variables were described using averages (based on the median values) or proportions with their respective 95% confidence intervals (95% CI). To establish whether there were differences in consumption by sex, the chi-squared test and t-tests for the equality of means (at the 95% level) were used. Ratio-based inequality indices, disparity, disproportionality, regression models, and entropy were calculated using Epidat, version 4.2 [29]. Calculations to establish the prevalence and frequency of consumption incorporated the sample design and were performed using Stata version 14 software (Stata Corporation, College Station, TX, USA) [30] and the original databases that generated the official ENSIN-2010 report [25]. The databases were provided by the ICBF, which is responsible for the ENSIN in Colombia.

#### Calculated inequality indices

Details regarding the theoretical and mathematical concepts that underlie the inequality index calculation, such as its scope and limitations, can be found in the Epidat version 4.2 help module [29] and many other specialized and easily accessible texts [21, 29, 31].

### Institutional review board

The authors declare that all procedures that contributed to this work comply with the ethical standards of the Declaration of Helsinki, revised in 2008. The research ethics committee of the National Institute of Health of Colombia approved the survey protocol, and all participants provided informed consent. The health research ethics committee of the Universidad Industrial de Santander states that anonymized data analyses are exempt from review.

## Results

Poverty in Bogotá, the capital of Colombia, was 13.1%; in Chocó, located on the Pacific coast, it was 64.0%. The average rate of monetary poverty for the country at the time of ENSIN-2010 was 41.5% (95% CI, 35.7 to 47.4). The mean prevalence of SB consumption among children aged 5 to 17 years was 88.5% (95% CI, 85.8 to 91.3); among girls, it was 87.1% (95% CI, 84.0 to 90.2), *p* = 0.271. In adult men between 18 and 64 years of age, the mean prevalence of consumption was 79.2% (95% CI, 75.7 to 82.8); in women, it was 70.7% (95% CI, 66.0 to 75.4), *p* < 0.0001. In children, the average median frequency of consumption in times/day was 0.35 (95% CI, 0.27-0.42); among girls, it was 0.40 (95% CI, 0.33 to 0.46), *p* = 0.187. In adult males, the mean frequency of consumption was 0.30 (95% CI, 0.24 to 0.35); among women, it was 0.20 (95% CI, 0.16 to 0.24), *p* < 0.002. The prevalence and the median frequency/day per geodemographic unit, age group and sex are presented in a table (Additional file 1). For all age groups and both sexes, the Spearman correlation (rs) of the mean prevalence and the average median SB consumption was high, ranging from 0.35 (adult females) to 0.68 (adult males).

### Rank-based inequality

The indices based on the ranges calculated here are a group of indicators based either on the relationship of the nutrition indicator to the extreme groups of a population hierarchy ordered according to a socioeconomic indicator or on the same nutrition indicator. The highest ratio was for 6 times/day in adult females, and the lowest rate ratio was 0.57 times/day in children. For both children and adults, regardless of gender, the mean prevalence and times/day for the country were lower than those estimated in geodemographic unit with lower monetary poverty. Consequently, negative values in the population are reported as absolute and relative attributable prevalences (Tables 1 and 2). In absolute terms, between 40% and 56% of the differences in SB consumption between geodemographic units in Colombia can be attributed to poverty conditions.

**Table 1:** Indices of inequality of SB consumption in children 5-17 years of age based on monetary poverty. Colombia, ensin-2010^a^

Ordered by socioeconomic variable^b^	Male		Female	
	Prevalence^c^	Times/day^d^	Prevalence^c^	Times/day^d^
Based on ranges. *n* = 24^e^				
Reason for extreme rates	1.01	0.57	1.02	1.00
Difference of extreme rates	0.50	−0.23	1.70	0.00
Prevalence attributable to population	−5.23	−0.20	−4.30	−0.16
Prevalence attributable to population (%)	−5.93	−56.39	−4.85	−40.84
Based on disparity or dispersion. *n* = 33				
Pearcy-Keppel	0.42	1.32	0.27	1.64
Pearcy-Keppel (Adjusted _A_)	26.48	0.20	20.19	0.24
Variance between groups (VEG)	43.50	0.03	59.52	0.02
Variance between groups (VEG_A_)	0.49	0.07	0.67	0.06
Based on disproportionality and concentration. *n* = 24				
Gini	0.04	0.24	0.05	0.21
Concentration	0.02	0.10	0.01	0.08
Based on regression models. *n* = 24				
Coefficient β	−0.07	−0.00	0.03	−0.00
Coefficient of determination	0.13	0.01	0.03	0.00
Inequality of slope	8.85	0.22	5.67	0.18
Inequality of Pamuk or relative	0.10	0.63	0.06	0.48
Inequality of Kunst and Mackenbach	1.10	1.45	1.06	1.39
Dimensional inequality	0.91	0.54	0.94	0.64
Based on the concept of entropy. *n* = 33				
Kullback-Liebler (Z)	0.00	0.10	0.00	0.08
Hoover or dissimilitudes (Z)	0.03	0.09	0.04	0.08
Theil (Z)	0.00	0.03	0.00	0.02

^a^Based on a Food Frequency Questionnaire, applied in the National Nutrition Survey in Colombia, 2010 (ENSIN-2010)

^b^For all calculations, the direction of the economic and health variable was negative

^c^Based on the average prevalence of consumption (%)

^d^Based on the median frequency of consumption (times/day)

^e^*n* is 24 when the monetary poverty data, which are not available for nine geodemographic units, are necessary for the calculation of the index

**Table 2:** indices of inequality of SB consumption in adults between 18 and 64 years of age based on monetary poverty. Colombia, ensin-2010^a^

Ordered by socioeconomic variable^b^	Male		Female	
	Prevalence^c^	Times/day^d^	Prevalence^c^	Times/day^d^
Based on ranges. *n* = 24^e^				
Reason for extreme rates	1.06	1.26	1.26	6.00
Difference of extreme rates	4.53	0.11	17.16	0.45
Prevalence attributable to population	−3.44	−0.13	1.69	−0.07
Prevalence attributable to population (%)	−4.39	−44.00	2.49	45.28
Based on disparity or dispersion. *n* = 33				
Pearcy-Keppel	0.57	2.29	0.66	1.23
Pearcy-Keppel (Adjusted _A_)	28.02	0.21	25.34	0.08
Variance between groups (VEG)	60.14	0.02	105.35	0.01
Variance between groups (VEG_A_)	0.77	0.05	1.55	0.05
Based on disproportionality and concentration. *n* = 24				
Gini	0.05	0.22	0.08	0.25
Concentration	0.01	0.12	−0.01	−0.05
Based on regression models. *n* = 24				
Coefficient β	0.12	−0.00	0.24	0.00
Coefficient of determination	0.00	0.00	0.02	0.11
Inequality of slope	3.23	0.22	−3.16	−0.05
Inequality of Pamuk or relative	0.04	0.73	0.05	0.28
Inequality of Kunst and Mackenbach	1.04	1.53	1.05	1.33
Dimensional inequality	3.23	0.49	1.04	1.29
Based on the concept of entropy. *n* = 33				
Kullback-Liebler (Z)	0.01	0.09	0.01	0.11
Hoover or dissimilitudes (Z)	0.04	0.15	0.06	0.17
Theil (Z)	0.01	0.08	0.01	0.11

^a^Based on a Food Frequency Questionnaire, applied in the National Nutrition Survey in Colombia, 2010 (ENSIN-2010)

^b^For all calculations, the direction of the economic and health variable was negative

^c^Based on the average prevalence of consumption (%)

^d^Based on the median frequency of consumption (times/day)

^e^*n* is 24 when the monetary poverty data, which are not available for nine geodemographic units, are necessary for the calculation of the index

### Inequality based on disproportionality and concentration

**Fig. 1:**
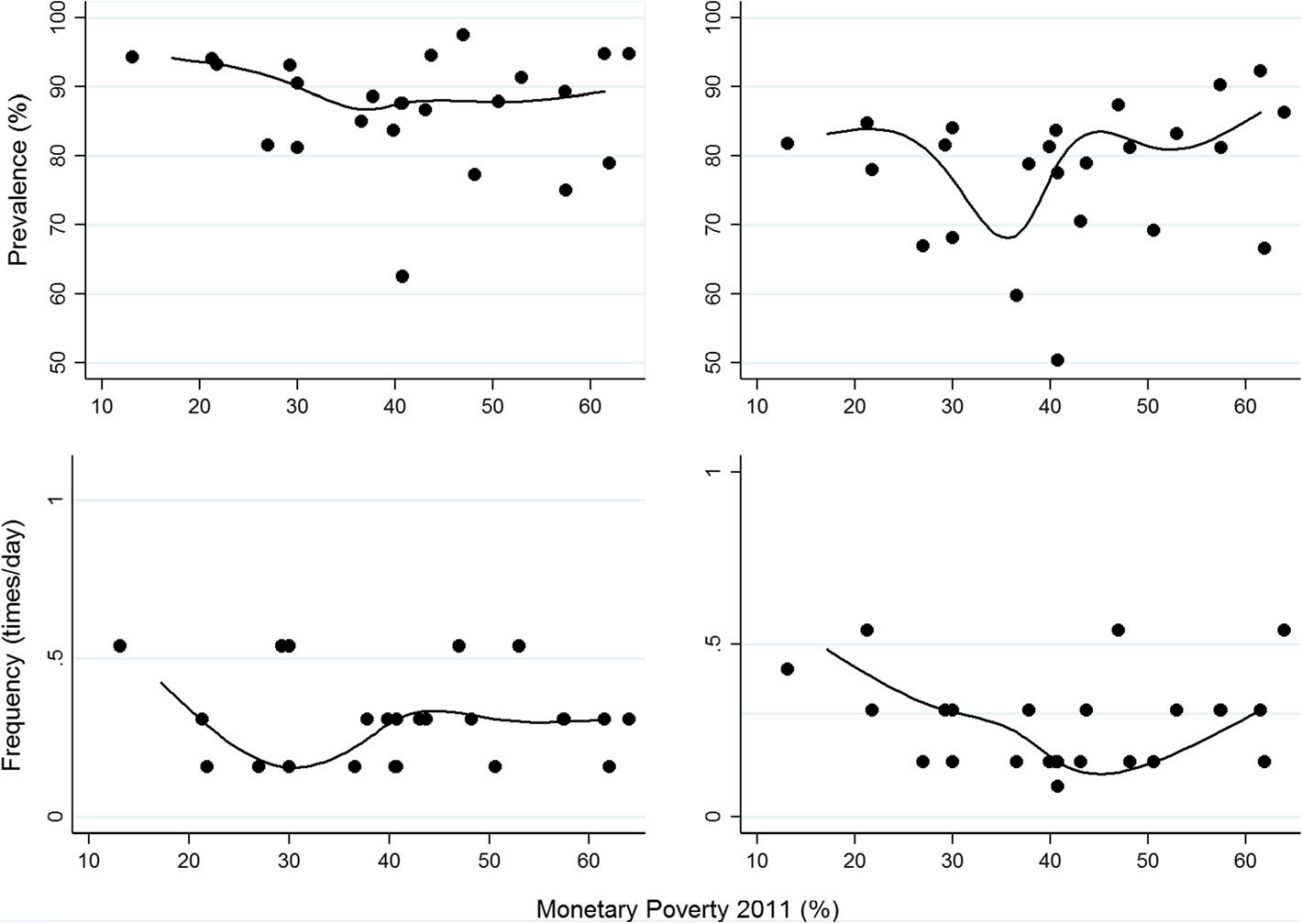
Consumption of sweetened beverages in males according to age and monetary poverty in the geodemographic units of Colombia, 2011. Relationship between monetary poverty and the two studied variables in the 33 geodemographic units in Colombia; the prevalence (%) and the average frequency (times/day) of consumption of sweetened beverages

**Fig. 2:**
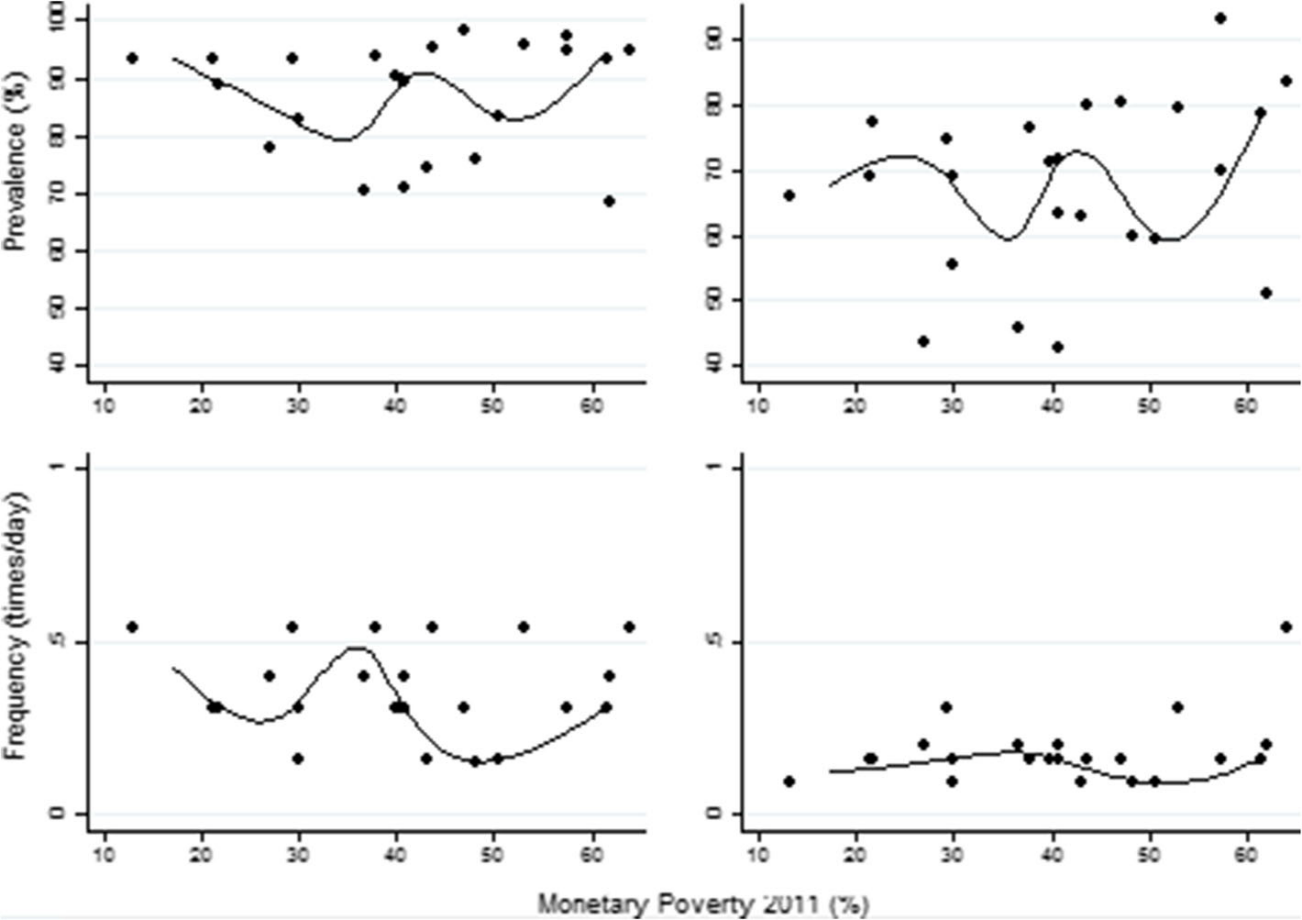
Consumption of sweetened beverages in females according to age and monetary poverty in the geodemographic units of Colombia, 2011. Relationship between monetary poverty and the two studied variables in the 33 geodemographic units in Colombia; the prevalence (%) and the average frequency (times/day) of consumption of sweetened beverages

The Gini coefficient is perhaps the best-known measure of inequality. It has values between 0 and 1. In this context, zero would indicate that SBs were consumed at all monetary poverty levels, that is, equality; in contrast, a value of 1 would represent a situation of perfect inequality, in which SBs were consumed only by people at the poverty level. Values less than 0.30 are considered high or almost perfect equality. The Gini coefficient and the concentration coefficient show that SB consumption inequality was low, with a prevalence ranging from 0.01 in minors to 0.08 in adults. The averages of the median frequency ranged from 0.08 in minors to 0.25 in adults (Tables 1 and 2). When the concentration indices and regression-based indices were plotted, there were relatively flat lines between poverty, mean prevalence, and the average median frequency of times/day of consumption (Figs. 1 and 2).

### Inequality based on regression models

In minors, it was demonstrated that as poverty increases in the geodemographic units, the average prevalence of consumption decreases between 7% and 0%. The relationship between consumption frequency and monetary poverty is non-existent. In adults, the behavior is similar. The inequality indices based on the concepts of entropy and disparities are presented in Tables 1 and 2.

### Inequality based on the concept of entropy

For this set of indices, values of zero (Z) indicate that there is no inequality. In this case, there is a perfect distribution of SB consumption. The values ranged from 0.01 to 0.17 (Tables 1 and 2).

## Discussion

Research on inequalities in health and, in this case, nutrition starts from an assumption that the most vulnerable - the poor, the displaced, or those belonging to minority groups - are disadvantaged and have lower health or nutrition conditions relative to the less disadvantaged [21–24, 29, 31, 32]. While this is generally true, it is not always the case in Colombia when nutritional variables are studied. When the gaps in prevalence and frequency/day of extreme consumption are compared, the absolute and relative inequality results based on ranges clearly show results consistent with the theoretical expectations for both age and sex: higher SB consumption was observed in the poorest geodemographic units. However, the absolute and relative population-attributable risks for frequency/day in male children (Table 1) and the prevalence for adult females (Table 2) indicate that in the richest geodemographic units, consumption was higher than the population mean, contrary to theoretical expectations. Rank-based indices are the most commonly used approaches in studies examining the inequality of health and nutrition variables [21, 29]. However, they have limitations; namely, they assess differences between extreme rates, but they do not describe what happens to the inequality in geodemographic units between the extreme ranges. Another limitation is that only weighted indices consider the size of the population within the geodemographic units [21, 29].

In addition, as reported here, the results based on the indices of disparity, concentration, regression models and entropy show how the prevalence and average frequency of SB consumption is slightly higher in the population with a higher monetary income or, in the best case, show no inequality according to the level of monetary poverty in the geodemographic units. Another indirect examination of the inequality of SB consumption in the Colombian population also showed that there is no inequality in the classical sense; this finding was related to the pattern of snack food consumption [32]. The main measures taken to reduce the consumption of sugary drinks have focuses on SB, for example, by limiting bottle sizes [33] or regulating or even prohibiting the sale of SB at school stores to make consumption difficult [14–16]. SB consumption taxes are justified by the excess weight of the population and its role in health events associated with chronic disease [6, 34, 35]. Economic inequality in SB consumption is an additional and useful aspect of discussions related to taxes on consumption [22–24]. In Colombia, where there is no evident economic inequality in SB consumption and where two brands control the market [36], the price-demand relationship for one of the brands was relatively inelastic during the 2002-2007 period, but since 2008, the price-demand relationship became elastic due to a price war related to the introduction of a new brand; this phenomenon was also observed in Peru, where it led to a situation in which an increase in income or a decrease in the cost of SB increased consumption [37]. This leads to new challenges in studying inequality in SB consumption because if inequality can be linked to a brand, brand loyalty or even a type of sweetener, then the inequality would be dynamic and at least in theory, it would be possible to establish a relationship between inequality in SB consumption and the price-demand elasticity of these characteristics. Although the inequality demonstrated here is low, a change in market strategies, price policies, bottle size or general inequality in Colombia, which is a vast country and one of the largest on the continent [3], can change the result when this exercise is repeated to consider SB consumption.

Research on inequalities in nutritional variables is scarce, particularly in the case of SB consumption, which makes it difficult to compare these results. Inequalities in SB consumption due to gender, ethnicity, or parental education may be related to socioeconomic and cultural status [20, 35]. No gender difference in consumption was observed here (Tables 1 and 2), and by design, it is only possible to make inferences regarding the economic inequality income proxy.

### Limitations of the study

Several aspects that warrant special care in analyzing these results are related to the measurement of SB consumption: a) the FFQ used in ENSIN-2010 did not specifically ask about the type of beverage consumed, the brand, or the type of sweetener used. In Colombia, according to industry and Euromonitor International data, the market for sugar-free and artificially sweetened beverages is growing rapidly; it reached 30% of the market share in 2014; b) The manner in which “soft drink and soda” consumption was measured did not differentiate between carbonated and uncarbonated soft drinks; and c) FFQs are simplified methods that can lead to memory bias and dilution of the association by reverse causality [38]. All these factors are limitations in the measurement of exposure and pose challenges for future ENSINs and other studies that attempt to measure SB consumption. These limitations can be overcome by a) including specific modules for the study of SB consumption in the surveys: These modules should incorporate the types of products through which the industry adapts to social pressure from increasingly informed consumers, including sugary drinks as well as sugar-free/artificially sweetened/flavored drinks, etc.; b) establishing better classifications for SB and SSB by incorporating additional characteristics, such as whether they include added nutritional components, such as fiber or micronutrients, and whether they are artificial or mixed with natural juices; and c) improving the discriminant capacity of the items in FFQ checklists and, when the studies are based on 24-h dietary recall, improving the description and coding of these foods.

The characterization of SB consumption by sex and age group in the geodemographic units of Colombia raised some methodological challenges for future measurements. This study established that in Colombia, there is no economic, age or sex inequality in SB consumption among geodemographic units. An understanding of SB consumption as an expression of the current stage of nutritional and food transitions is necessary to ensure that public policies and preventive practices are coherent.

## Conclusions

In conclusion, in Colombia, there is no inequality in the consumption of SB. The study of inequalities in nutritional variables (in this case, the consumption of SB), indicates that the poorest members of the population are not always those with the greatest nutritional risk. Discussions regarding the appropriateness of consumption taxes must consider the following questions: Who consumes the most SB, and who will pay the taxes: the rich or the poor? The imposition of taxes on SB consumption in societies where most of the population is poor and where there is no evidence of inequality in consumption can be regressive. The study of the consumption of SB should incorporate greater specificity, since the speed at which the industry changes the presentation and components of SB make consumption difficult to measure. A high prevalence of consumption does not necessarily mean a high frequency of consumption; when studying SB, it is necessary to have a complete picture, including the prevalence and frequency of consumption and the quantity consumed. The results of this research, which was based on population surveys and included both children (5-17 years) and adults (18-64 years), will provide scientific evidence allowing decision makers to supplement available information on SB consumption, to assess the relevance of their policies and to evaluate the results of these policies as these data provide a baseline against which to evaluate the impact of measures and interventions. It is necessary to address the methodological challenges of measuring SB consumption amid the nutritional and food transition that is evident in Latin America and in middle-income countries such as Colombia.

Finally, the finding that there is no economic inequality in SB consumption by age or sex exemplifies the different dynamics involved in diet. In developing countries, inequalities in dietary consumption do not always exist; their presence depends on the food studied and the social and economic context, which are summarized here in terms of geography.

## Additional file

Additional file 1: Table S1. Contains the data on the prevalence of consumption and the median frequency (times / day) of the consumption of sweetened beverages by age group and sex in each geodemographic unit studied. (DOCX 20 kb)

## Abbreviations

DANE: *Departamento Administrativo Nacional de Estadística* (National Administrative Department of Statistics)
ENSIN: Colombian National Nutrition Survey
FFQ: Food Frequency Questionnaire
ICBF: The Colombian Family Welfare Institute
SB: Sweetened Beverages
SSB: Sugar-Sweetened Beverages

## References

[1] Ocampo TPR, Prada GGE, Herrán FOF (2014). Patrones de consumo alimentario y exceso de peso infantil; encuesta de la situación nutricional en Colombia, 2010. Rev Chil Nutr.

[2] Herrán OF, Patiño GA, Del Castillo SE (2016). Dietary transition and excess weight in adults according to the Encuesta de la Situación Nutricional en Colombia, 2010. Biomedica.

[3] Departamento Nacional de Planeación (DPN) (2011). Índice de Pobreza multidimensional para Colombia (Ipm-Colombia) 1997-2010.

[4] Jansen EC, Herrán OF, Villamor E (2015). Trends and correlates of age at menarche in Colombia: results from a nationally representative survey. Econ Hum Biol.

[5] Popkin BM, Adair LS, Ng SW (2012). Global nutrition transition and the pandemic of obesity in developing countries. Nutr Rev.

[6] Popkin BM (2001). Nutrition in transition: the changing global nutrition challenge. Asia Pac J Clin Nutr.

[7] de Blanco ML, Carmona A (2005). La transición alimentaria y nutricional: un reto en el Siglo XXI. An Venez Nutr.

[8] Moreno-Altamirano L, Hernández-Montoya D, Silberman M, Capraro S, García-García JJ, Soto-Estrada G (2014). The nutrition transition and the double burden of malnutrition: changes in dietary patterns 1961-2009 in the Mexican socioeconomic context. Arch Latinoam Nutr.

[9] Kasper NM, Herrán OF, Villamor E (2014). Obesity prevalence in Colombian adults is increasing fastest in lower socio-economic status groups and urban residents: results from two nationally representative surveys. Public Health Nutr.

[10] Swinburn B, Egger G, Raza F (1999). Dissecting obesogenic environments: the development and application of a framework for identifying and prioritizing environmental interventions for obesity. Prev Med.

[11] Mazarello Paes V, Hesketh K, O’Malley C, Moore H, Summerbell C, Griffin S (2015). Determinants of sugar-sweetened beverage consumption in young children: a systematic review. Obes Rev.

[12] Bowry AD, Lewey J, Dugani SB, Choudhry NK (2015). The burden of cardiovascular disease in low- and middle-income countries: epidemiology and management. Can J Cardiol.

[13] Bucher Della Torre S, Keller A, Laure Depeyre J, Kruseman M (2016). Sugar-sweetened beverages and obesity risk in children and adolescents: a systematic analysis on how methodological quality may influence conclusions. J Acad Nutr Diet.

[14] Rodríguez-Burelo MR, Avalos-García MI, Ramón CL (2014). Consumo de bebidas de alto contenido calórico en México: un reto para la salud pública. Salud Tabasco.

[15] Castaño DO. Impuesto a Gaseosas en Colombia: Postobón Responde. El Colombiano; 2016. http://m.elcolombiano.com/impuesto-a-gaseosas-en-colombia-postobon-responde-GF4502019. Accessed 18 Jan 2017.

[16] Jou J, Techakehakij W (2012). International application of sugar-sweetened beverage (SSB) taxation in obesity reduction: factors that may influence policy effectiveness in country-specific contexts. Health Policy.

[17] Rehm CD, Matte TD, Van Wye G, Young C, Frieden TR (2008). Demographic and behavioral factors associated with daily sugar-sweetened soda consumption in new York City adults. J Urban Health.

[18] Dodd AH, Briefel R, Cabili C, Wilson A, Crepinsek MK (2013). Disparities in consumption of sugar-sweetened and other beverages by race/ethnicity and obesity status among United States schoolchildren. J Nutr Educ Behav.

[19] Guerrero-López CM, Unar-Munguía M, Colchero MA (2017). Price elasticity of the demand for soft drinks, other sugar-sweetened beverages and energy dense food in Chile. BMC Public Health.

[20] van Ansem WJ, van Lenthe FJ, Schrijvers CT, Rodenburg G, van de Mheen D (2014). Socio-economic inequalities in children's snack consumption and sugar-sweetened beverage consumption: the contribution of home environmental factors. Br J Nutr.

[21] Wagstaff A, Paci P, van Doorslaer E (1991). On the measurement of inequalities in health. Soc Sci Med.

[22] Pearcy JN, Keppel KG (2002). A summary measure of health disparity. Public Health Rep.

[23] Regidor E (2004). Measures of health inequalities: part 1. J Epidemiol Commun Health..

[24] Regidor E (2004). Measures of health inequalities: part 2. J Epidemiol Commun Health.

[25] Instituto Colombiano de Bienestar Familiar. Encuesta nacional de la Situación Nutricional en Colombia; 2010. http://www.icbf.gov.co/portal/page/portal/Descargas1/Resumenfi.pdf. Accessed 15 Nov 2014.

[26] Bautista LE, Herrán OF, Pryer JA (2005). Development and simulated validation of a food-frequency questionnaire for the Colombian population. Public Health Nutr.

[27] Herrán OF, Ardila Lizarazo MF (2006). Validity and reproducibility of two semi-quantitative alcohol frequency questionnaires for Colombian population. Public Health Nutr.

[28] Departamento Administrativo Nacional de Estadística (DANE), Pobreza y Desigualdad; 2011. https://www.dane.gov.co/index.php/estadisticas-por-tema/pobreza-y-condiciones-de-vida/pobreza-y-desigualdad/pobreza-y-desigualdad-2011#pobreza-monetaria-por-departamentos-2011. Accessed 8 Nov 2016.

[29] de Sanidade C, Xunta de Galicia. España, Organización Panamericana de la Salud (OPS-OMS), Universidad CES C. Epidat: Programa para Análisis Epidemiológico de datos; 2016. http://www.sergas.es/Saude-publica/EPIDAT.

[30] StataCorp (2015). Stata statistical software: release 14.

[31] Mackenbach JP, Kunst AE (1997). Measuring the magnitude of socio-economic inequalities in health: an overview of available measures illustrated with two examples from Europe. Soc Sci Med.

[32] Herrán OF, Patiño GA, DelCastillo SE (2015). Desigualdad y nutrición: Encuesta de la situación nutricional en Colombia, 2010. Rev Bras Saude Mater Infant.

[33] Mantzari E, Hollands GJ, Pechey R, Jebb S, Marteau TM (2015). Impact of bottle size on in-home consumption of sugar-sweetened beverages: protocol for a feasibility and acceptability study. Pilot Feasibility Stud.

[34] Hu FB, Malik VS (2010). Sugar-sweetened beverages and risk of obesity and type 2 diabetes: epidemiologic evidence. Physiol Behav.

[35] Pearson-Stuttard J, Bandosz P, Rehm C, Penalvo J, Whitsel L, Gaziano T (2016). Reduction of cardiovascular disease inequalities in the USA through dietary policy. Lancet.

[36] López LSM, Echavarría AJG. La estructura del Mercado y la inversión extranjera directa en la industria de bebidas no alcohólicas en Colombia (2002-2009): Los casos de Coca Cola, Big Cola y la empresa nacional Postobón. Universidad De La Salle; 2011. file:///H:/Documents and Settings/trabajo año 2016/Articulo gaseosa/Desigualgaseoa/Tesis Elasticidad Gaseosas.pdf. Accessed 13 Feb 2017.

[37] Suarez-Rojas PD. El Mercado de Bebidas Gaseosas en el Perú. El Caso Añaños-Kola REAL – Monografiascom; 2007. http://www.monografias.com/trabajos35/mercado-gaseosas-peru/mercado-gaseosas-peru.shtml. Accessed 13 Feb 2017.

[38] Willett W (2013). Nutritional epidemiology.

