# Supplementary material for: Consumption of sweetened-beverages and poverty in Colombia: when access is not an advantage
Source: BMC Public Health. 2018 Jan 15;18:136. doi: 10.1186/s12889-018-5037-1 (PMC5769354; doi:10.1186/s12889-018-5037-1)
Supplement: Additional file 1: Table S1. — Contains the data on the prevalence of consumption and the median frequency (times / day) of the consumption of sweetened beverages by age group and sex in each geodemographic unit studied. (DOCX 20 kb) [file 12889_2018_5037_MOESM1_ESM.docx]

| **Table 1 Supplementary Material-On Line** prevalence (%) and median frequency (times/day)^a^. Consumption of Sweetened-Beverages. Colombia. ENSIN-2010 | | | | | | | | | |
| --- | --- | --- | --- | --- | --- | --- | --- | --- | --- |
| Geodegraphic unit | 5 to 17 y | | | | 18 to 64 y | | | | |
|  | Males | | Females | | Males | | Females | | |
|  | Prevalence  (%) | times/d | Prevalence  (%) | times/d | Prevalence  (%) | times/d | Prevalence  (%) | | times/d |
| Antioquia | 93.10 | 0.54 | 93.00 | 0.54 | 81.53 | 0.31 | 74.49 | | 0.31 |
| Atlántico | 88.50 | 0.31 | 93.60 | 0.54 | 78.80 | 0.31 | 76.50 | | 0.16 |
| Bogotá. D.C. | 94.20 | 0.54 | 93.00 | 0.54 | 81.76 | 0.43 | 66.09 | | 0.09 |
| Bolívar | 94.50 | 0.31 | 95.20 | 0.54 | 78.91 | 0.31 | 80.03 | | 0.16 |
| Boyacá | 83.70 | 0.31 | 90.20 | 0.31 | 81.31 | 0.16 | 71.34 | | 0.16 |
| Caldas | 85.00 | 0.16 | 70.20 | 0.40 | 59.79 | 0.16 | 45.86 | | 0.20 |
| Caquetá | 87.60 | 0.31 | 89.50 | 0.31 | 77.51 | 0.16 | 63.51 | | 0.16 |
| Cauca | 78.90 | 0.16 | 68.50 | 0.40 | 66.56 | 0.16 | 50.93 | | 0.20 |
| Cesar | 97.50 | 0.54 | 98.40 | 0.31 | 87.40 | 0.54 | 80.22 | | 0.16 |
| Córdoba | 94.70 | 0.31 | 93.20 | 0.31 | 92.25 | 0.31 | 78.67 | | 0.16 |
| Cundinamarca | 94.00 | 0.31 | 93.00 | 0.31 | 84.68 | 0.54 | 68.83 | | 0.16 |
| Chocó | 94.70 | 0.31 | 94.70 | 0.54 | 86.29 | 0.54 | 83.25 | | 0.54 |
| Huila | 77.30 | 0.31 | 76.10 | 0.15 | 81.15 | 0.16 | 59.80 | | 0.09 |
| La Guajira | 89.30 | 0.31 | 94.60 | 0.31 | 90.24 | 0.31 | 92.85 | | 0.16 |
| Magdalena | 75.00 | 0.31 | 97.30 | 0.31 | 81.22 | 0.31 | 69.87 | | 0.16 |
| Meta | 90.40 | 0.54 | 82.70 | 0.31 | 84.04 | 0.31 | 68.79 | | 0.16 |
| Nariño | 87.80 | 0.16 | 83.10 | 0.16 | 69.23 | 0.16 | 59.33 | | 0.09 |
| N. de Santander | 87.60 | 0.16 | 89.60 | 0.31 | 83.70 | 0.16 | 71.51 | | 0.16 |
| Quindío | 62.50 | 0.16 | 70.90 | 0.40 | 50.44 | 0.09 | 42.68 | | 0.20 |
| Risaralda | 81.50 | 0.16 | 77.70 | 0.40 | 66.88 | 0.16 | 43.80 | | 0.20 |
| Santander | 93.20 | 0.16 | 88.80 | 0.31 | 77.97 | 0.31 | 77.11 | | 0.16 |
| Sucre | 91.30 | 0.54 | 95.70 | 0.54 | 83.23 | 0.31 | 79.40 | | 0.31 |
| Tolima | 86.70 | 0.31 | 74.50 | 0.16 | 70.51 | 0.16 | 63.00 | | 0.09 |
| Valle del Cauca | 81.20 | 0.16 | 82.60 | 0.16 | 68.15 | 0.16 | 55.41 | 0.09 | |
| Arauca | 96.30 | 0.54 | 96.50 | 0.85 | 97.13 | 0.54 | 89.90 | 0.54 | |
| Casanare | 91.70 | 0.31 | 92.50 | 0.31 | 82.07 | 0.54 | 73.48 | 0.16 | |
| Putumayo | 79.40 | 0.15 | 80.50 | 0.31 | 73.63 | 0.15 | 68.44 | 0.16 | |
| San Andrés^b^ | 96.40 | 0.15 | 93.80 | 0.85 | 89.25 | 0.15 | 81.14 | 0.54 | |
| Amazonas | 91.60 | 0.31 | 92.10 | 0.31 | 85.14 | 0.31 | 86.04 | 0.16 | |
| Guainía | 86.30 | 0.15 | 82.30 | 0.16 | 66.20 | 0.15 | 61.78 | 0.09 | |
| Guaviare | 99.40 | 0.85 | 90.00 | 0.54 | 87.71 | 0.54 | 86.34 | 0.16 | |
| Vaupés | 96.80 | 1.09 | 85.10 | 0.85 | 90.11 | 0.54 | 87.76 | 0.31 | |
| Vichada | 93.90 | 0.54 | 74.00 | 0.31 | 79.17 | 0.31 | 75.18 | 0.16 | |
| ^a^ Based on a Food Frequency Questionnaire | | | | | | | | | |
| ^b^ Includes Providencia and Santa Catalina islands. | | | | | | | | | |
